# Haemorrhagic Occlusive Retinal Vasculitis Following Cataract Surgery Without Intraocular Vancomycin

**DOI:** 10.7759/cureus.114716

**Published:** 2026-08-18

**Authors:** Sarah Grech, Alastair Bezzina, Andrea Calleja

**Affiliations:** 1 Ophthalmology, Mater Dei Hospital, Msida, MLT; 2 Internal Medicine, Mater Dei Hospital, Msida, MLT

**Keywords:** case report, cataract extraction, haemorrhage, optical coherence, retinal vasculitis, vancomycin

## Abstract

This paper reports a rare case of haemorrhagic occlusive retinal vasculitis (HORV) following cataract surgery in the absence of intraocular vancomycin, raising important considerations about alternative aetiologies and perioperative management. A 79-year-old male patient with a background of type 2 diabetes, benign prostatic hyperplasia and asthma experienced recurrent hypersensitivity reactions to topical mydriatics during preoperative assessments for right eye cataract surgery in the private sector. Surgery was ultimately performed without topical mydriatics; full mydriasis was achieved with the use of intracameral mydriatic agents and iris hooks. No intraocular vancomycin was administered. Postoperative care included standard antibiotic-steroid eye drops. Three days postoperatively, the patient presented with sudden, painless vision loss. Examination revealed extensive retinal haemorrhages, vasculitis, and macular ischaemia. Imaging confirmed widespread retinal vascular damage. Infectious and autoimmune screens were negative. A diagnosis of HORV was made, and the patient was treated with intravenous methylprednisolone, topical corticosteroids, and later sub-tenon triamcinolone. Partial visual improvement was observed. This case highlights the possibility of HORV in the absence of vancomycin, suggesting that other immune-mediated or hypersensitivity pathways, in response to different triggers, may contribute to its pathogenesis.

## Introduction

Haemorrhagic occlusive retinal vasculitis (HORV) is a rare but devastating sequela to otherwise routine intraocular surgery, which was historically associated with the use of intraocular vancomycin injection at the end of phacoemulsification surgery [1]. It is presumed to be secondary to hypersensitivity to pharmacological agents used during cataract surgery in the first eye, which then causes a florid and often sight-threatening retinal vasculitis and haemorrhage in the second eye. Similar adverse reactions have also been observed with the use of intravitreal anti-vascular endothelial growth factor agents such as brolucizumab and faricimab [2]. The main consequence of HORV is the onset of ischaemia, which results in irreversible damage to the retinal parenchyma, which in turn may lead to neovascular sequelae, including bleeding, tractional vitreo-retinopathy and neovascular glaucoma. To the best of our knowledge, this is the first case report showcasing HORV in the context of intra-cameral mydriatic use in a patient with a history of repeat hypersensitivity reactions to the same agents, namely phenylephrine and cyclopentolate.

## Case presentation

We report the case of a 79-year-old male with a medical history of type 2 diabetes mellitus, benign prostatic hyperplasia, and asthma and no prior reported drug allergies. The patient underwent an uneventful elective cataract extraction in the left eye in February 2024. No intraocular vancomycin was used at any stage. Subsequently, he was scheduled for cataract surgery in the right eye. However, on three separate occasions in the private sector, administration of topical 1% tropicamide, 1% cyclopentolate, and 10% phenylephrine led to the rapid development of a discharge, marked conjunctival injection, and mild papillary reaction. These findings were attributed to hypersensitivity to the mydriatic agents, prompting cancellation and rescheduling of the surgery each time. Despite being thoroughly counselled on the potential increased risk of postoperative complications, the patient elected to proceed.

A modified preoperative regimen was implemented, including a brief course of topical povidone-iodine and tobramycin/dexamethasone drops. On the day of surgery, topical mydriatics were omitted, and adequate pupillary dilation was achieved using intracameral 2.5% phenylephrine, 0.5% cyclopentolate, and mechanical iris hooks. Intracameral cefuroxime (1 mg/0.1 mL) was administered intraoperatively at the end of surgery. The procedure proceeded uneventfully, and the patient was continued on tobramycin/dexamethasone postoperatively.

On first-day review, the patient exhibited mild swelling of the right upper eyelid, conjunctival chemosis, and mild injection. Posterior segment examination was unremarkable.

**Figure 1:**
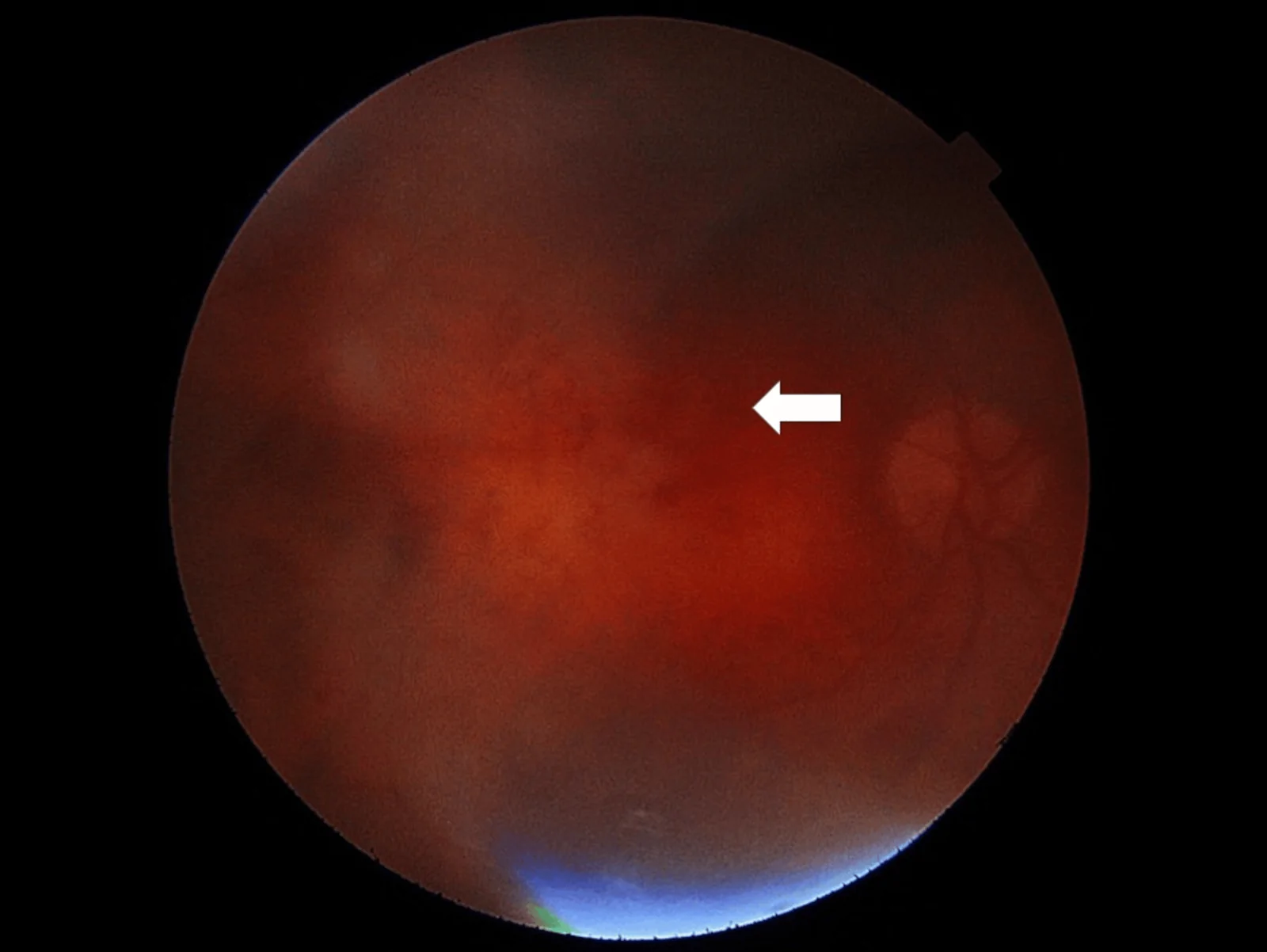
Fundal photo of the right eye demonstrating vitreous haze (causing poor image clarity) as well as multiple haemorrhages within the posterior pole area (arrow).

Three days postoperatively, the patient presented to the ophthalmic emergency clinic with a sudden, painless reduction in vision in the right eye from the previous evening. Snellen visual acuity was reduced to counting fingers at close to the face. Ocular examination revealed conjunctival injection and mild anterior chamber inflammation with a flare but no hypopyon. No corneal oedema was evident. Fundus examination disclosed a mild-to-moderate vitreous haze and extensive retinal haemorrhages in all four quadrants (Figure 1), arteriolar and venular sheathing, and patchy retinal pallor. The patient’s left eye was unremarkable. On further questioning, the patient confirmed the use of multiple antimicrobial agents for other maladies, including the use of second-generation cephalosporin, cefaclor, without experiencing any adverse reactions. No evidence of intraocular inflammation was observed or documented during the postoperative period following his first cataract surgery.

A vitreous tap was performed in order to exclude a viral cause for the presentation, and samples were sent for bacterial and viral cultures. Polymerase chain reaction testing for Epstein-Barr virus, cytomegalovirus, varicella-zoster virus, and herpes simplex virus was negative. Serological work-up, including antinuclear antibody, antineutrophil cytoplasmic antibody, toxoplasma antibodies, syphilis, and human immunodeficiency virus, was also negative.

**Figure 2:**
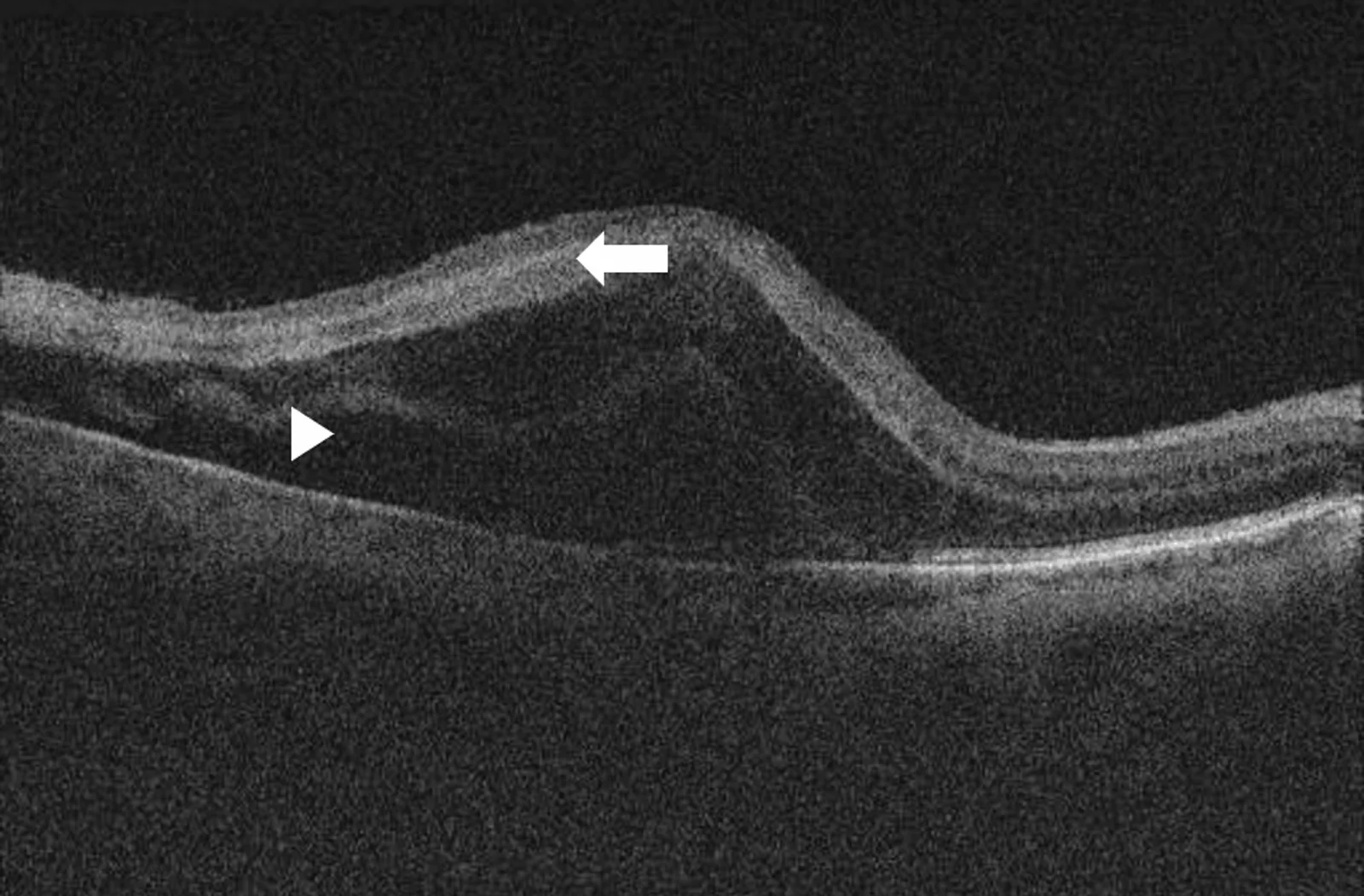
Initial optical coherence tomography showing inner retinal hyper-reflectivity (arrow) and diffuse outer retinal oedema (arrowhead) of the right macula.

**Figure 3:**
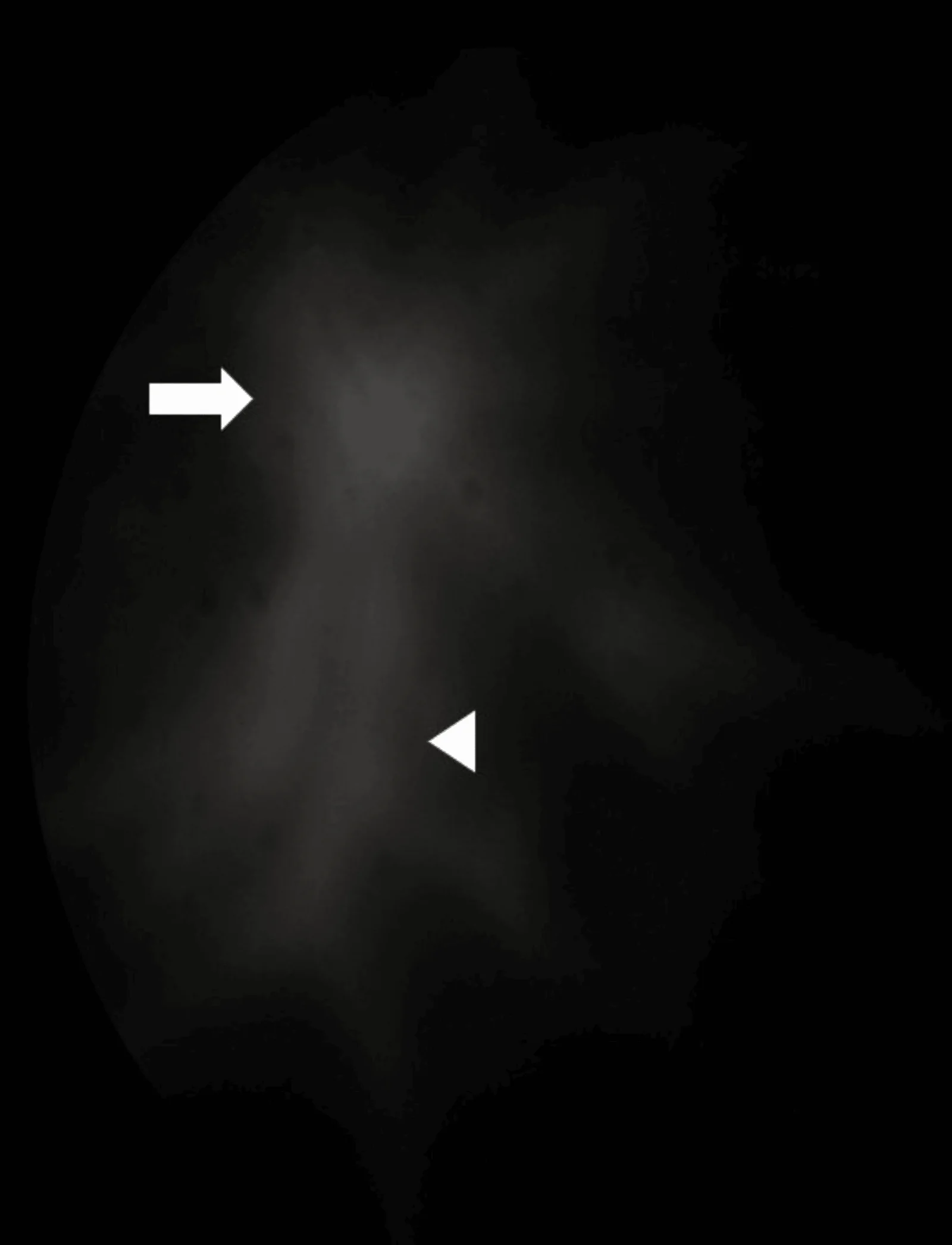
Marked fluorescein leakage from the optic nerve head (arrow) and around the proximal vascular arcades (arrowhead). Note: Image blurring is caused by the vitreous haze on presentation.

Optical coherence tomography (OCT) of the macula (Figure 2) revealed significant central macular oedema with associated inner retinal hyperreflectivity. Intravenous fluorescein angiography revealed diffuse vascular leakage despite the significant haze posed by the associated vitritis (Figure 3). These investigations were performed to assess for areas of retinal non-perfusion and damage.

Based on clinical and imaging findings, a presumptive diagnosis of HORV was made. The patient was admitted and commenced on high-dose intravenous methylprednisolone (1 gram daily for three days), along with topical 0.1% dexamethasone four times a day, 0.3% gentamicin four times a day, and 1% atropine once daily.

Following initiation of therapy, the patient’s vision improved to counting fingers at 3 metres, with no improvement on pinhole testing. A sub-tenon injection of triamcinolone acetonide (40 mg/1 mL) was administered inferonasally without complications. He was discharged on oral prednisolone (70 mg daily), 1% atropine daily, and 0.1% dexamethasone drops.

**Figure 4:**
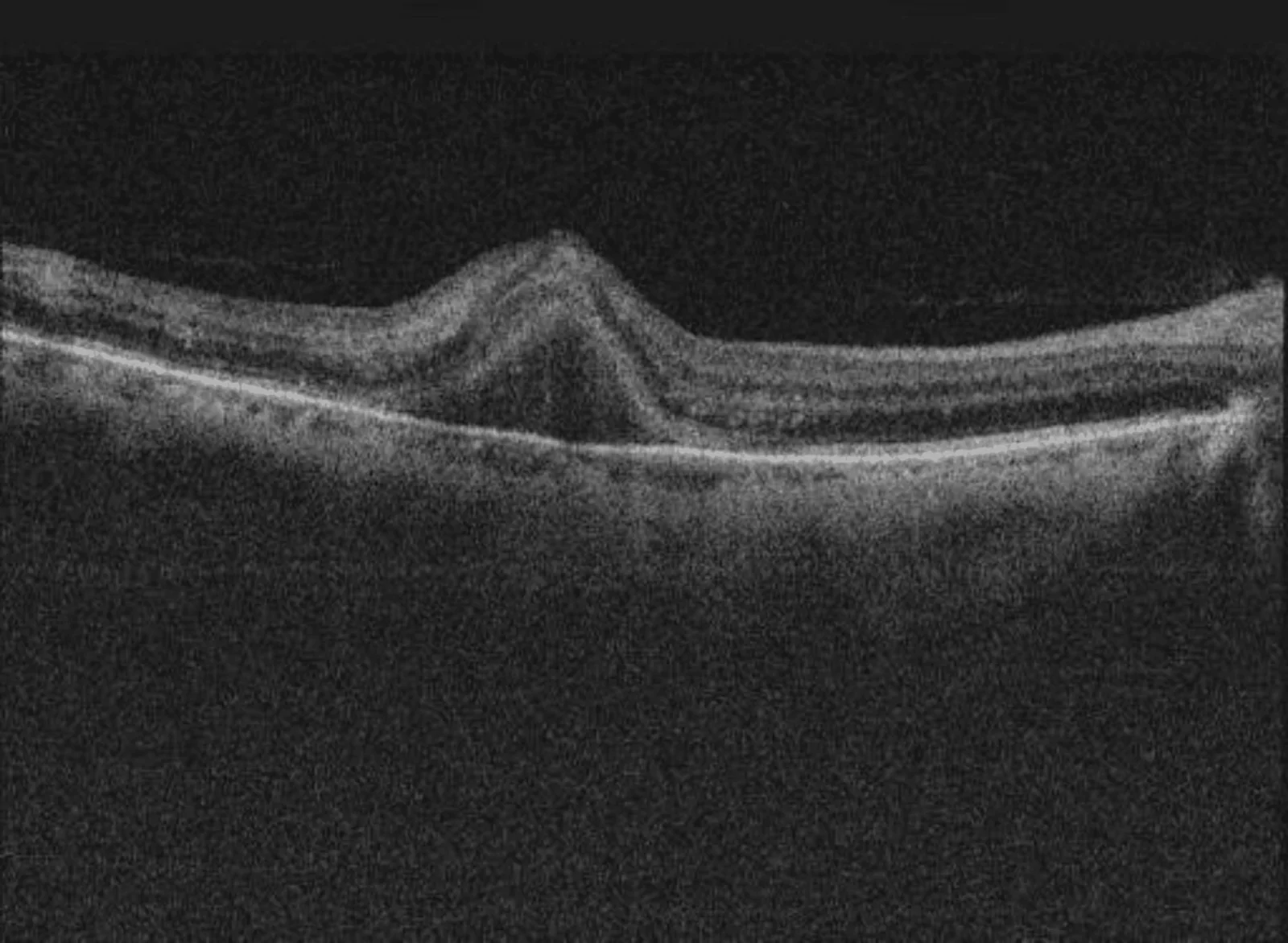
Optical coherence tomography of the macula of the right eye nine days following methylprednisolone pulsing and sub-tenon triamcinolone injection followed by an oral prednisolone regime. A marked reduction in macular oedema and inner retinal reflectivity can be observed when compared to Figure 1.

**Figure 5:**
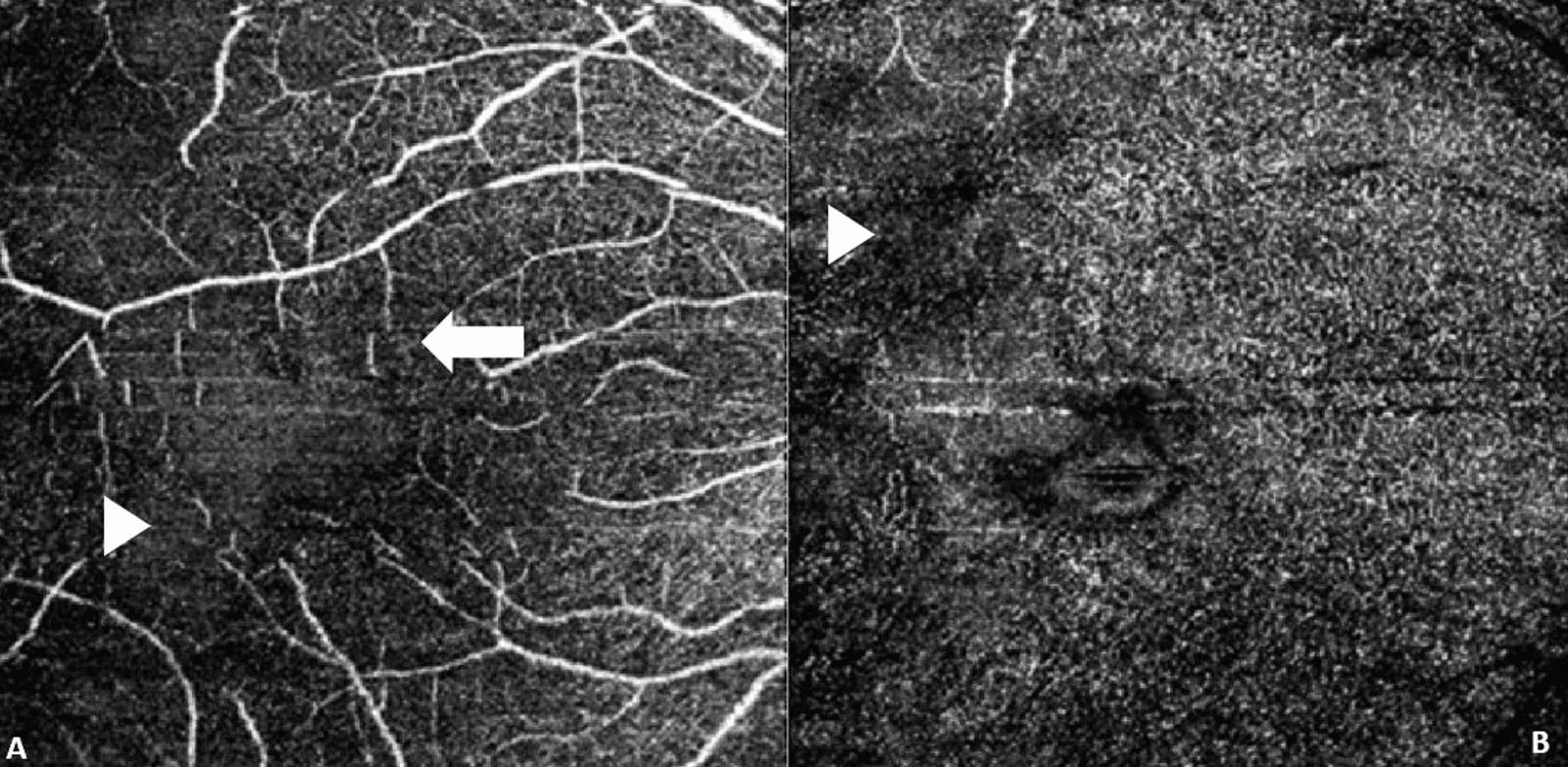
Macular optical coherence tomography angiography (6x6) of the right eye demonstrating vessel pruning (arrow) and flow voids (arrowhead) in the superficial vascular complex (Pane A) and flow voids (arrowhead) in the deep vascular complex (Pane B).

Upon review nine days later, his visual acuity had remained the same while the conjunctival injection had started to abate. Posterior segment examination revealed persistent segmental vascular constriction with improvement in vascular sheathing and a decrease in macular pallor. A repeat OCT showed a reduction in central macular thickness (Figure 4), but significant flow voids and vessel pruning were noted in the macular area on OCT angiography (Figure 5). Prednisolone was started being taillisted down, with a plan to decrease by 10 mg per week.

**Figure 6:**
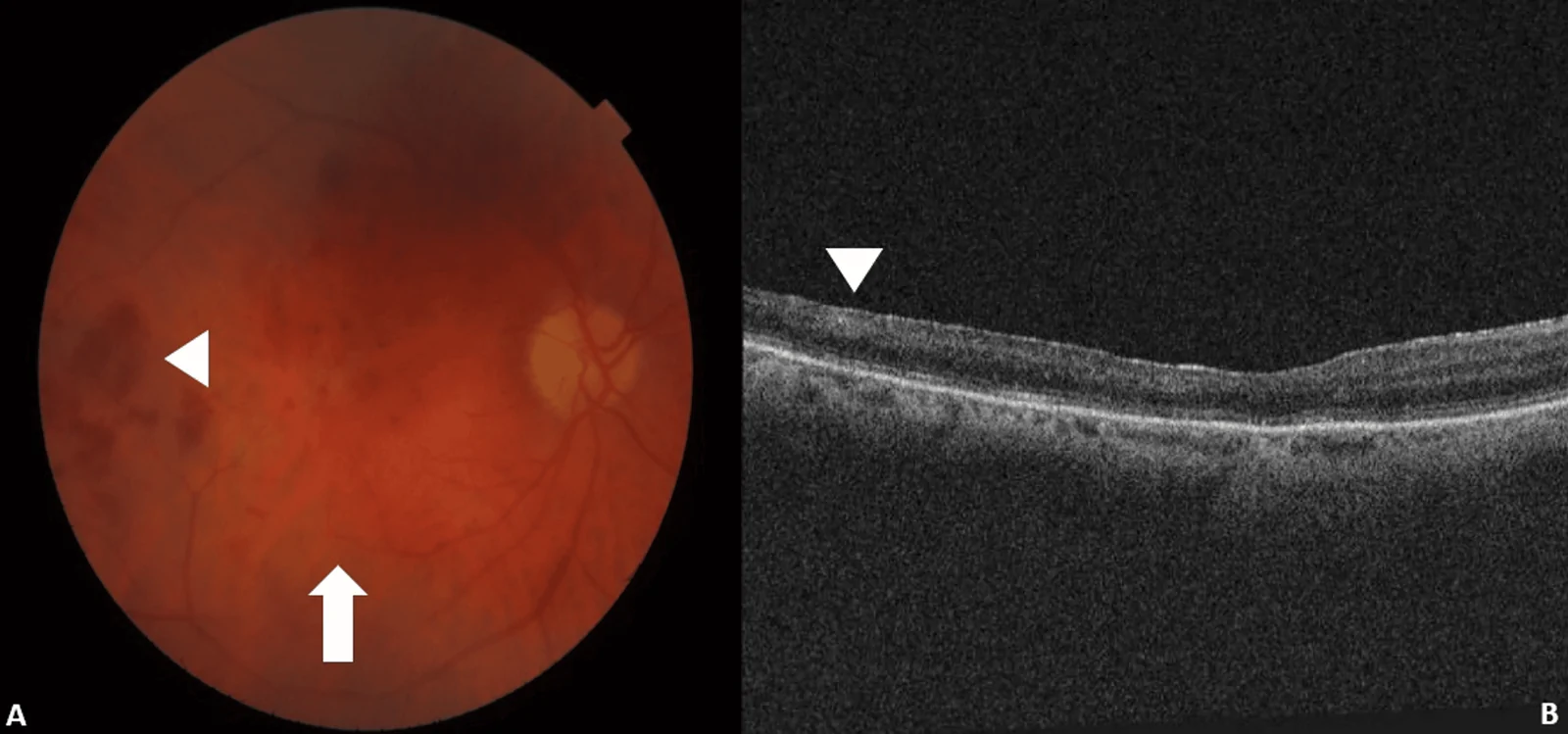
Colour fundal photo of the right eye eight weeks following initiation of corticosteroid therapy (Pane A) showing an improvement in vitreous haze as well as vessel attenuation (arrow) and residual intra-retinal haemorrhages (arrowhead). Pane B (right) demonstrates a macular optical coherence tomography (OCT) during the same eighy-week follow-up review exhibiting full resolution of the macular oedema and inner retinal hyper-reflectivity but with ensuing macular atrophy and disruption of the inner retinal layers in the temporal and foveal areas (arrowhead).

He was reviewed eight weeks post-operatively, where no improvement in visual acuity was noted despite remaining on oral prednisolone. Posterior segment examination revealed persistent blot and dot haemorrhages, while OCT showed minimal residual intra-retinal fluid (Figure 6). Oral prednisolone was tapered off completely, and the patient was scheduled for frequent reviews to assess for any sequelae related to the posterior segment ischaemia.

## Discussion

Postoperative HORV is one of the rare inflammatory sequelae of phacoemulsification surgery, which has been described following the advent of intraocular antibiotic use, namely intra-cameral vancomycin, after the European Society of Cataract and Refractive Surgeons published its landmark study confirming the importance of using intracameral antibiotics to reduce the risk of postoperative endophthalmitis [3]. Similar issues have also been described following the use of intravitreal anti-vascular endothelial growth factor agents such as broculizumab and the bivalent monoclonal antibody faricimab [2]. To the best of our knowledge, this case report describes the first documented case of HORV suspected to be secondary to the use of intracameral mydriatic agents, namely cyclopentolate and phenylephrine, following uncomplicated cataract surgery. The ocular surface reaction to the topical mydriatic agents noted in the patient involved in this case report, without a history of similar symptomatology prior to phacoemulsification surgery in his first eye, suggests sensitisation of the aforementioned agents from prior use, thus supporting the theory of delayed hypersensitivity behind this phenomenon. The use of cephalosporin antimicrobial therapy following his first phacoemulsification as well as systemically during the previous years, along with the observed reaction to the topical mydriatics, tips the likelihood towards the mydriatic agents being the main offending agent in this case.

The events and disease phenotype described in this case report conform with the diagnostic criteria set up by Witkin et al. in their case series of vancomycin-related HORV [1], namely: The event occurred after routine and uneventful phacoemulsification surgery with no evidence of posterior segment abnormalities within 24 hours following surgery. The patient presented on postoperative day 3 complaining of sudden-onset painless loss of vision in the operated eye, suggestive of a retinal vascular phenomenon. The visual acuity on presentation was down to detection of hand movements, reflecting a severe degree of ischaemia. Moderate vitreous inflammation, denoted by the presence of 2+ vitreous cells and 1-2+ haze (Standardization of Uveitis Nomenclature (SUN) criteria [4]), was noted on presentation. Both arteriolitis and periphlebitis were noted on fundoscopy with associated peri-vascular haemorrhages. Profuse perivascular leakage was noted on fluorescein angiography with surrounding capillary dropout associated with patchy superficial and deep capillary plexus flow voids on OCT-A and inner retinal hyper-reflectivity of OCT B-scan confirming inner retinal ischaemia.

The main differential diagnoses for this presentation would be endophthalmitis; however, the patient denied pain, and there was no evidence of florid posterior uveitis or viral retinitis. However, all viral samples were negative, and the timing after cataract surgery makes this diagnosis less likely compared to HORV and central retinal vein occlusion (CRVO); however, no venular dilatation was noted on examination and investigation of this patient.

The pathophysiology of post-operative HORV is still being debated, but in view of the latency of the reaction, albeit short, direct drug toxicity is unlikely in such cases, as such reactions would happen immediately following administration of the offending agent [5, 6]. Conflicting reports can be found in the literature, with the majority of documented cases having been described in the light of intraocular vancomycin use at the end of the procedure [1, 6-8], pointing to a type III hypersensitivity reaction, similar to that observed in vancomycin-induced leukocytoclastic vasculitis [1, 9]. On the other hand, histopathological analysis of enucleated eyes in patients who suffered from HORV, described in a later publication, has demonstrated a widespread haemorrhagic vasculopathy with associated endothelial proliferation and multifocal thrombosis and a non-granulomatous choroidal inflammation with a predominance of CD4 and CD8 positive T-cells, thus suggesting the possibility of a type IV hypersensitivity reaction. Of note, there was no evidence of a leukocytoclastic reaction in the retinal vasculature reported, as suggested by Witkin et al. [1], in the former analysis [5].

The treatment of HORV remains mostly empirical, namely the use of corticosteroid therapy (systemic as well as intraocular or periocular) and the use of anti-vascular endothelial growth factor agents and pan-retinal photocoagulation if retinal ischaemia or its sequelae are identified [1,10]. In our patient, treatment with pulsed intravenous methylprednisolone followed by oral prednisolone in conjunction with sub-tenon triamcinolone yielded minimal visual improvement, which could be related to the late presentation following the onset of symptoms, allowing for the development of extensive ischaemic damage, thus underlining the importance of prompt treatment in such cases.

## Conclusions

This case highlights the severe consequences of HORV and the need for prompt review and management in patients presenting with sudden and painless loss of vision following phacoemulsification surgery or other procedures involving intraocular drug injection perioperatively. A detailed examination with close attention to the presence of retinal haemorrhages and vasculitis, which in turn prompt further investigation with OCT and fluorescein angiography, is required in order not to miss this rare but potentially sight-threatening condition. HORV mimics, such as viral retinitis and endophthalmitis, must be excluded so as not to worsen a potentially treatable condition.

Notably, this case occurred in the absence of intraocular vancomycin administration, challenging the current understanding of HORV pathophysiology and raising the possibility of alternative triggers or immune-mediated mechanisms. Early identification is crucial in order to institute treatment and maximise the chances of sight recovery. More research into the pathophysiology of this serious condition is required in order to guide treatment options and regimes, which are as yet empiric and poorly evidence-based.
